# Editorial: Brain dopaminergic mechanisms

**DOI:** 10.3389/fnsyn.2023.1292511

**Published:** 2023-09-27

**Authors:** Ben Yang, Roman A. Romanov, Jinbin Xu, Jean-Pierre Mothet

**Affiliations:** ^1^Department of Neuroscience, Feinberg School of Medicine, Northwestern University, Chicago, IL, United States; ^2^Center for Brain Research, Medical University of Vienna, Vienna, Austria; ^3^Division of Radiological Sciences, Department of Radiology, Mallinckrodt Institute of Radiology, Washington University in St. Louis, St. Louis, MO, United States; ^4^Laboratoire LuMin UMR9024, Université Paris-Saclay, ENS Paris-Saclay, CNRS, CentraleSupelec, Gif-sur-Yvette, France

**Keywords:** dopamine, Parkinson's disease, basal ganglia (BG), spiny projection neurons, cholinergic interneurons of the striatum, morphine, impulsive compulsive behaviors, glucagon like peptide 1 (GLP-1)

Dopamine was first synthesized in 1910. It was initially named 3-hydroxytyramine and gained little interest in the science community. About a half-century later, neuroscientists started to realize that dopamine is a neurotransmitter and plays a key role in Parkinson's disease (PD). It was then formally renamed “dopamine” by Sir Henry Dale at the Physiology Society Meeting at Cambridge (Hornykiewics, 1986). Now, more than a century after its discovery, dopamine remains the key player in brain control of motor functions, activity state, reward, and drug addiction and is tightly incorporated in multiple interplay circuits. In this Research Topic, we collected research articles investigating dopamine-related mechanisms of regulating neuronal properties and potentially PD, as well as non-dopaminergic modulation of such regulations at the level of the basal ganglia interplay. Likewise, review articles discuss the dopamine-related basal ganglia circuits and network determinants of motor disability in PD.

In PD, midbrain dopaminergic neurons innervating the basal ganglia are lost. This results in an increased neuronal activity, particularly exaggerated beta oscillations, in the basal ganglia (Deffains and Bergman, 2019). In the basal ganglia's input nucleus, the striatum, the hyperactivity is generally thought to be due to hyperactivity in spiny projection neurons (SPNs) that consist of 95% of cells in the striatum. However, Padilla-Orozco et al. found it is not the SPNs, but the cholinergic interneurons (CINs) that are the main source of hyperactivity in dopamine-depleted striatum. This consists with decades of clinical observations that when striatal dopamine level drops, acetylcholine level rises, and anticholinergic drugs were used to treat the motor symptoms of PD in the pre-levodopa era. The authors further showed that changes in both intrinsic and synaptic properties contribute to CIN hyperactivity. Among the synaptic inputs, not only glutamatergic, but also GABAergic and nicotinic transmission drive the pathological hyperactivity in CINs. These different mechanisms that all contribute to CIN hyperactivity during parkinsonism provide new directions for future investigations on basal ganglia hyperactivity and therapeutic targets for treating PD.

Other than motor symptoms, PD patients also suffer non-motor symptoms, including sleep disorders, speech problems, fatigue, pain, anxiety, depression, hallucination, etc. Dopamine medication can also induce impulsive-compulsive behaviors (ICBs), exacerbating incentive motivation and choice impulsivity. To better understand the neurocognitive mechanisms underlying ICBs in PD, Dawson et al. performed a deep evaluation of impulsivity in PD patients with and without ICBs. The authors confirmed that PD patients with ICBs show an exacerbation of incentive motivation and choice impulsivity. Specifically, they have a higher tendency to choose hard tasks regardless of reward probability and have a high level of delay discounting. The authors also examined antisocial behaviors and found greater reactive aggression in PD patients with ICBs. The authors concluded that a transdiagnostic neurocognitive endophenotype approach should be adopted to understand and predict the addictive and aggressive behaviors in PD patients under dopamine medication.

The cardinal motor symptoms of PD are caused by the loss of midbrain dopaminergic neurons. Alzheimer's disease (AD), another common neurodegenerative disorder, is characterized by the loss of glutamatergic and cholinergic neurons in the forebrain as well as other regions. Growth factors that have neural protective effects in principle should protect neuronal loss and maintain synaptic transmission. A key issue with most growth factors is the ability to cross the blood-brain barrier. Hölscher reviewed studies on glucagon-like peptide 1 (GLP-1) and glucose-dependent insulinotropic peptide (GIP) receptor agonists that are ready to cross the blood-brain barrier for the protection of PD and AD. In PD mouse models, GLP-1 and GIP receptor agonists enhanced dopamine levels in the striatum, protected dopaminergic neuronal loss, and improved motor functions. Similarly, these agonists also enhanced synaptic transmission, protected synaptic plasticity, and facilitated learning and memory in AD mouse models. Various clinical trials of different GLP-1 and GIP agonists have also shown protective effects in PD and AD patients. Ongoing and future trials, particularly of dual receptor agonists, are promising in developing effective and safe treatments for PD and AD. This is even more exciting given the recent FDA approval of dual receptor agonists tirzepatide that is superior to GLP-1 agonist semaglutide in treating diabetes at much higher doses but with similar mild to moderate side effects (Frías et al., 2021).

Dopamine release is spatiotemporally regulated by multiple mechanisms, including tonic and phasic, axonal and somatodendritic, somatic activity-driven and distal activity-driven mechanisms. Recent studies suggest Nogo receptor 1 (NgR1) may also regulate dopamine release. The article of Arvidsson et al. used both NgR1-overexpression and NgR1 knockout mice to study how NgR1 affects dopamine release. The authors found NgR1 overexpression reduced dopamine release while NgR1 knockout increased dopamine release. While dopamine may induce global synaptic plasticity changes via broad dopaminergic projections, NgR1 modules local structural changes. This work suggests a mechanism where neuronal activity temporally down-regulates NgR1 expression, which leads to an increase in dopamine release, together these create a window where local and global synaptic plasticity regulating systems may work in synergy for learning and memory consolidation.

The functional circuit organization of the basal ganglia consists of two major pathways initiating from the striatal SPNs. The direct pathway SPNs project directly to the basal ganglia output structures, the substantia nigra pars reticulata and globus pallidus interna; the indirect pathway SPNs project indirectly to the output structures via the globus pallidus externa and the subthalamic nucleus. Interestingly, it was found that the direct pathway SPNs express type 1 dopamine receptors (D1Rs) while the indirect pathway SPNs express type 2 dopamine receptors (D2Rs). However, this conclusion did not come easily. Gerfen wrote an excellent historical review on this topic where he played the central role in not just demonstrating the receptor expression patterns in these two pathways, but also creating the first mouse lines labeling these two pathways that are most commonly used in the basal ganglia field now. Gerfen also integrated more recent findings that have challenged the classical “go/no-go” model of the direct and indirect pathways and proposed a more complex circuit organization of the basal ganglia, particularly at the globus pallidus externa.

Dopamine-induced changes in the nucleus accumbens are crucial for reward learning and drug addiction. In turn, McDevitt et al. investigated how morphine, a highly addictive opioid, alters the synaptic and intrinsic properties of the dopamine cellular targets: D1R- and D2R-expressing SPNs in the nucleus accumbens shell. They found morphine treatment did not alter the synaptic and intrinsic properties in D1R-SPNs. However, morphine treatment reduced the frequency of both the miniature excitatory and inhibitory postsynaptic currents in D2R-SPNs. Interestingly, they found that as the frequency of the miniature postsynaptic currents decreased, there's a concomitant increase in D2R-SPN intrinsic excitability. Notably, the functional output of synaptically-driven firing in D2R-SPNs was unchanged, suggesting a potential mechanism for homeostatic modulation of neuronal activity in D2R-SPNs upon morphine exposure.

Another recent study led by the Surmeier group also challenged the classical model with their finding that striatal dopamine depletion is not sufficient to induce PD motor symptoms, but dopamine depletion throughout the basal ganglia is (Gonzalez-Rodriguez et al., 2021). Surmeier et al. therefore raised eight problems with the classical model and provided detailed discussion on (1) the striatal circuit with an emphasis on the role of cholinergic modulations, (2) striatal adaption in PD focusing on how dopamine depletion-induced synaptic plasticity changes affect SPN ensembles essential for motor functions, (3) dopamine release beyond the striatum, particularly en passant release in nuclei downstream of striatum and dendritic release in substantia nigra pars reticulata.

More than a century has passed since the initial synthesis of dopamine. Still more is going to be discovered regarding dopamine's function in health and diseases. We hope you enjoy reading this Research Topic and we look forward to new advances in the dopamine field.

## Author contributions

BY: Writing—original draft, Writing—review and editing. RR: Writing—review and editing. JX: Writing—review and editing. J-PM: Writing—review and editing.
